# Allozyme variability in populations of trout (Salmo trutta) from the rivers of Russia and Iran

**DOI:** 10.1186/2193-1801-1-48

**Published:** 2012-11-20

**Authors:** Vahid Taghizadeh, Gerigori Genadevich Novikov, Abdolreza Jahanbakhshi

**Affiliations:** 1 Department of Fishery, Faculty of Fisheries and Environment, Gorgan University of Agricultural Science and Natural Resources, Gorgan, Iran; 2 Biological Faculty, Moscow State University, Vorob’evy gory, Moscow, 117234 Russia

**Keywords:** Allozyme, Enzyme, Russia, Iran, Trout

## Abstract

For the first time, an analysis was carried out of allozyme variability in trout (Salmo trutta) from three rivers of Iran. We studied 23 gene loci coding enzymes: glycerol-3-phosphate dehydrogenase (G3PDH), aspartate aminotransferase (AAT), malate dehydrogenase (MDH), lactate dehydrogenase (LDH), creatine kinase (CK), malic enzyme [NADP-dependent MDH] (MEP), superoxide dismutase (SOD), esterase (EST), and esterase D (EST–D). The obtained data demonstrate the similarity between the trout samples from different rivers of Iran according to genetic characteristics. Taking into account the differences by allozyme markers of allele frequencies and allele composition of some loci, we should expect that Iranian trout diverges significantly in genetics from the other trout populations of the Caspian Sea.

## Introduction

Trout (Salmo trutta) is distributed in a wide area covering a significant part of Europe, the western part of Asia, and the northern part of Africa (Berg 1948; Reshetnikov et al. 2002). Trout possesses a wide population differentiation, settles in different water bodies, and forms the range of ecological and geographical races. Trout of the Caspian Sea Basin belongs to one of the most valuable commercial fishes of the region, the commercial importance of which increases as the resources of salmon fishes decrease. Natural populations of trout are preserved in many rivers in the territory of Iran, but they are endangered because of a permanently increasing anthropogenic impact including mining (especially coal mining) in the northern part of Iran, where the main spawning rivers of this species are situated. The problems of protection and rational use of trout resources cannot be solved without accounting the population structure of this species. Simultaneously, monitoring of results of industrial activity becomes a more and more important general biological task.

The works on Iranian trout are few, have morphological–ecological character, and belong mostly to the beginning–middle of the last century (Derzhavin 1934; Kozhin 1957). The study of this species carried out currently in Iran has mostly a commercial orientation because of the development of aquaculture (Abdoli 1994; Abdollahi 1995; Sharifi 2000). The data on genetic characteristics of trout from the rivers of Iran are practically absent.

The goal of this work was formulated as the study of allozyme variability and the features of genetic differentiation in trout populations from the rivers of the Caspian coast of Iran.

## Material and method

Material was collected in 2011 and 2012 in the territory of Iran in the Chalus, Karganrud (“wild” populations), and Kharaz (“farm” population) rivers and from the Vorob’ev Brook (Kandalaksha Gulf, the White Sea) in the territory of Russia; the sizes of samples were, respectively, 50, 50, 93, and 41 specimens. The sample of trout from the White Sea Basin was taken as a reference sample to standardize used methods and obtained results because there was abundant available literature data on ecological, biological, and genetic characteristics of these trout (especially of trout from the Vorob’ev Brook) populations (Osinov 1984a, 1984b, 1988, 2004; Kuzishchin and Novikov 1994; Osinov and Bernatche, 1996; Kuzishchin 1997; Makhrov et al., 1998; Makhrov, 1999). Obtained results were analyzed using also available literature on trout from the rivers of the Caspian and Black sea basins.

**Figure 1:**
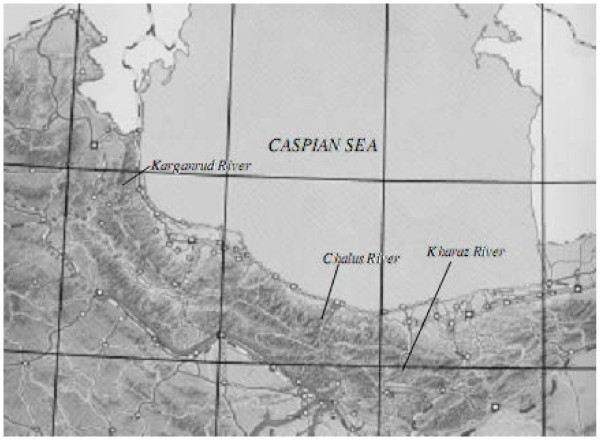
**Places of trout Salmo trutta sampling in Iranian rivers.**

The Karganrud, Kharaz, and Chalus rivers (Figure 1) run in the northern part of Iran in the Alborza mountain range and fall into the Caspian Sea. Hydrological and hydrochemical characteristics of these rivers are typical for mountain rivers. Maximal distance between these rivers is 420 km, so collecting of trout samples covered completely the Caspian coast of Iran.

Fishes were captured by means of electric fishing (excluding the fish farm on the Kharaz River) and were transported in a frozen state (whole) to the Department of Ichthyology, Moscow State University, for biological and genetic analysis.

Genetic analysis was carried out by marker loci used traditionally for trout by Russian and foreign researchers (Osinov, 1984b; Allendorf et al., 1977). Samples of the following tissues were taken for the analysis: white muscles, liver, eye. Electrophoresis in polyacrylamide gel was carried out according to two methods described in literature (Davis 1964; Peacock et al., 1965; Osinov, 1984a).

The gene loci were studied, coding following enzymes: glycerol-3-phosphate dehydrogenase(G3PDH), aspartate aminotransferase (AAT), malate dehydrogenase (MDH), lactate dehydrogenase (LDH), creatine kinase (CK), malic enzyme [NADP-dependent MDH] (MEP), superoxide dismutase (SOD), esterase (EST), and esterase D (EST–D). The alleles in the studied allozyme loci during the primary treatment of material were designated according to the nomenclature rules and manuals for salmon fishes (Osinov, 1984a, 1984b; Osinov and Bernatche, 1996; Allendorf et al., 1977; Allendorf and Utter, 1979; Waples, 1988; Shaklee et al., 1990). To increase the reliability of allele identification according to their relative electrophoretic mobility, we carried out the combined distillation of samples from the Caspian Sea and White Sea basins in the same block.

Genetic interpretation of observed variations in the studied loci was carried out according the schemes described in literature. Simple codominant heritability is assumed for the loci EST–D*, LDH–5*, sMEP–3*, EST–2*, mSOD–2*, and sSOD–3* (Osinov 1984b, 1988; Osinov and Pavlov 1998). The model of inheritance suggested the equality of frequencies for both loci in alleles and was suggested for isoloci sMDH–3,4* and sAAT–1,2* (Osinov, 1984b, 1988; Osinov and Bernatche 1996). The model of inheritance assuming the polymorphism of only one locus was suggested for duplicated loci mMEP–1,2*, sMDH–1,2*, LDH–1,2*, LDH–3,4*, G3PDH–2,3*, and CK–1,2* (Osinov, 1984b, 1988; Osinov and Bernatche 1996). The correspondence between observed and theoretical (according the Hardy-Weinberg-Castle model) distribution of genotypes was tested using the χ2 test. The significance of difference of allele frequencies between the samples was assessed with the help of Fisher’s F- test (Urbakh, 1963). Genetic identity and standard genetic distances between the trout populations were calculated according Nei’s method (Nei 1975a, 1975b, 1981) in the TEPGA software program package.

## Results and discussion

The population-genetic analysis we carried out using a sum total of 23 enzyme loci, 10 of which were polymorphic (Table 1).

**Table 1:** **Allele frequencies in polymorphic loci in the studied samples of trout Salmo trutta**

		Samples			
Locus, allel		Vorob’ev	Kharaz	Chalus	Karganrud
		Brook	River	River	River
*sAAT*-*1*,2*100		0.870	-	-	-
*120		0.220	-	-	-
	*n*	41	-	-	-
	χ*2*	16.25	-	-	-
	*p*	0.05			
*LDH*-5*100		0.351	0.984	0.96	0.97
*90		0.649	0.016	0.04	0.03
	*n*	37	93	50	50
	χ*2*	3.07	40.65	11.48	21.50
	*p*	0.05	0.001	0.01	0.001
*sMDH*-2*100		0.611	-	-	-
*142 (=*118)^*1*^		0.389	-	-	-
	*n*	36	-	-	-
	χ*2*	1.120	-	-	-
	*p*	0.05			
*sMDH*-*3*,4*100		1	0.864	0.835	0.923
*75(=*50*)^*1*^		0	0.136	0.923	0.076
	*n*	41	92	48	49
	χ*2*	0	6.86	2.23	3.22
	*p*		0.05	0.05	0.05
G3PDH-2*100		0.988	0.983	0.97	0.95
*116		0	0.017	0.03	0.05
*75		0.012	0	0	0
	*n*	41	91	50	40
	χ*2*	0.01	0.03	0.05	0.11
	*p*	0.05	0.05	0.05	0.05
sSOD-2*100		1	0	0	0
*80		0	0.296	0.194	0.2
*60		0	0.704	0.806	0.8
	*n*	41	93	49	45
	χ*2*	0	0.19	0.59	0.55
	*p*		0.05	0.05	0.05
sMEP-3*100		1	-	-	0.333
*90		0	-	-	0.667
	*n*	41	-	-	24
	χ*2*	0	-	-	0.38
	*p*				0.05
EST-D*100		1	0	0	0
*145		0	0.025	0	0.033
*124		0	0.975	1	0.667
	*n*	41	79	50	30
	χ*2*	0	0.05	0	0.04
	*p*		0.05		0.05

Note:^1^—according the nomenclature suggested by Osinov (1988); n—the number of studied fishes.

Testing of equilibrium (χ2-test) demonstrated good agreement between observed and theoretical distributions of genotypes in all samples excluding LDH–5* (in three samples from the rivers of Iran). Locus LDH–5* was present in the studied samples by the two-allele system: alternative allele *90 had high frequency in the sample from the White Sea Basin, but this allele was presented as rare in the samples from the Caspian Sea (Iranian rivers) Basin (Table 1). In the samples from every river (Kharaz, Chalus, and Karganrud), one homozygote 90/90 and single heterozygotes 100/90 (respectively 1, 2, and 1) were present in locus LDH–5*, and this caused the displacement of equilibrium and heterogeneity at a high level of significance. We cannot provide an explanation of this situation at the current stage, but it should be noted that similar character of phenotype distribution (presence in the single of heterozygotes and homozygotes by rare allele) we found in the study of allozyme markers of redfish Sebastes mentella from the Irminger Sea (Stroganov & Novikov 2004); the bias at the expense of the appearance of the homozygote of rare allele was observed sometimes in salmons, for example, in Dolly Varden trout Salvelinus malma (Osinov 2002). The significance of difference between the frequencies of the main alleles in the studied loci was evaluated (paired-comparison) using Fisher’s F-test Urbakh 1963). Significant differences in allele frequencies between the samples from Iranian rivers and the sample from the Vorob’ev Brook in the White Sea Basin was determined by five loci: LDH–5*, sMDH–3,4*, sSOD–2*, sMEP–3*, and EST–D* (Table 2). The samples from Iranian rivers did not differ significantly from one another in the frequencies of the main alleles in the studied loci.

**Table 2:** **Paired**-**comparison** (**by Fisher**) **of significance of differences between allele frequencies in the studied samples of trout Salmo trutta**

	Values of U-criterion					
Loci	Vorob’ev Brook	Vorob’ev Brook–	Vorob’ev Brook–	Kharaz River–	Kharaz River–	Chalus River–
	Kharaz River	Chalus River	Karganrud River	Chalus River	Karganrud River	Karganrud River
LDH-5*	8.108***	6.780***	6.780***	0.590	–	–
sMDH-3,4*	4.020***	3.930***	2.660**	0.454	1.090	1.350
G3PDH-2*	0.220	0.610	1.039	0.490	0.990	–
sSOD-2*	10.600***	10.500***	10.500***	1.350	1.230	0.029
sMEP-3*	–	–	9.030***	–	–	–
EST-D*	13.300***	14.800***	12.800***	1.760	0.139	1.580

Note: The difference is significant at p: * <0.05, ** <0.01, *** <0.001.

**Figure 2:**
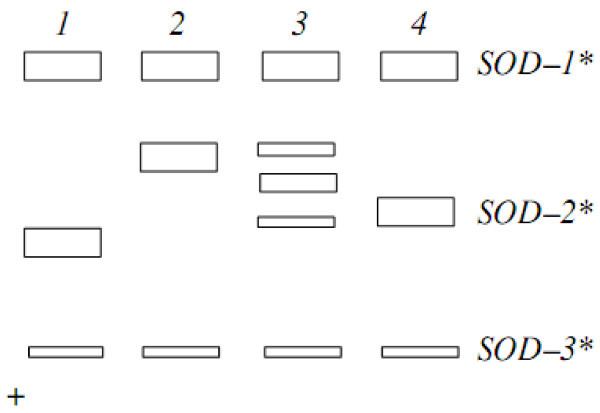
**Electrophoretic phenotypes of loci SOD**–**1**,**2**,3* **in muscle of trout Salmo trutta and their genetic interpretation.** Trout of White Sea: 1, SOD–1* (100/100), SOD–2* (100/100), SOD–3* (100/100). Trout of Iran: 2, SOD–1* (100/100), SOD–2* (60/60), SOD–3* (100/100); 3, SOD–1* (100/100), SOD–2* (60/80), SOD–3* (100/100); 4, SOD–1* (100/100), SOD–2* (80/80), SOD–3*(100/100).

**Figure 3:**
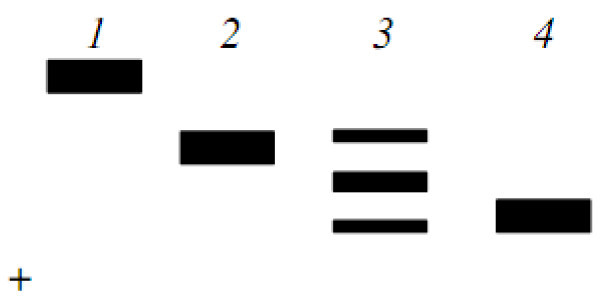
**Electrophoretic phenotypes of locus EST**–D* **in muscle of trout Salmo trutta and their genetic interpretation.** Trout of White Sea: 1, EST–D* (100/100). Trout of Iran: 2, EST–D* (124/124); 3, EST–D* (124/145); 4, EST–D* (145/145).

**Figure 4:**
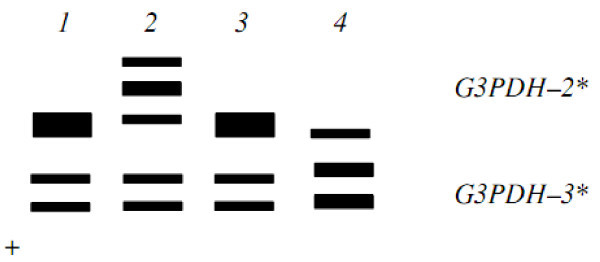
**Electrophoretic phenotypes of locus G3PDH**–**2**,3* **in muscle of trout Salmo trutta and their genetic interpretation.** Trout of White Sea: 1, G3PDH–2* (100/100); 2, G3PDH–2* (100/75). Trout of Iran: 3, G3PDH–2* (100/100); 4, G3PDH–2* (100/116).

The comparison of allele composition of gene loci, which is used for revealing the qualitative differences between the populations, was carried out using own data and involving the published results (Osinov 1984b, 1988; Kazakov & Titov 1992; Osinov & Bernatche 1996). The differences in allele composition between the samples from the basins of the White Sea and Caspian Sea were found by following studied enzyme loci: G3PDH–2*, sSOD–2*, EST–D* and sMEP–3* (Figures 2, 3 and 4). Allele composition of loci in the samples from the Caspian Sea Basin, i.e., within the group of Caspian trout, was also compared using available literature data.

The differences in allele composition between the sam- ples from Iranian rivers of the southern Caspian Sea coast (own data) and from the rivers of the western coast of the Caspian Sea (Osinov 1984b, 1988; Osinov & Bernatche 1996) were found by the following enzyme loci: EST–D*, G3PDH–2*, and sSOD–2*. The data by Osinov & Bernatche (1996) suggest also significant genetic differentiation of trout populations from the rivers of the northwestern and western coast of the Caspian Sea.

Genetic distances and identities of trout populations were calculated in our work by 12 gene loci. High values of identity of the samples from Iranian rivers were obtained (0.9968–0.9977). Standard genetic distances between trout samples from Iranian rivers ranged from 0.0023 to 0.0031, and this fact suggests their genetic community (Table 3).

**Table 3:** **Estimates of standard genetic distances** (**above the diagonal**) **and genetic identity** (**under the diagonal**) **calculated between the Iranian populations of trout Salmo trutta**

Rivers	Vorob’ev	Kharaz	Chalus	Karganrud
Vorob’ev	-	0.3217	0.3395	0.3237
Kharaz	0.7249	-	0.0023	0.0032
Chalus	0.7122	0.9977	-	0.0031
Karganrud	0.7235	0.9968	0.9969	-

Standard genetic distances in trout from the basins of the Caspian Sea (Iran) and the White Sea were com- pared also using available literature data. According to the results presented in the work of Osinov & Bernatchez (1996) by 36 allozyme loci, the values of genetic distances between the trout samples from the rivers of the Caspian Sea basin ranged from 0.026 to 0.068 (the sample from the Tsna River was not considered), and in the samples from the rivers of the White Sea Basin, from 0.0091 to 0.024. Genetic distances between the basins ranged from 0.0804 to 0.1607. According to our data, the comparison between the trout samples from the Vorob’ev Brook and from Iranian rivers revealed higher values of standard genetic distances: 0.3217– 0.3395. However, the values could be overestimated in our case because of less total number of analyzed loci, and this fact agrees well with Nei’s note (1981): average index of distortion increases when less than 30 loci are used in calculations.

Hence, the data obtained in our study demonstratethe similarity between trout samples from different Iranian rivers by genetic characteristics (allozyme mark- ers: allele frequencies and allele composition of loci). Taking into consideration that the Karganrud River is situated in the west and the Kharaz River almost in the easternmost Caspian coast of Iran, we can state with relative significance that Iranian populations of trout represent the group weakly differentiated genetically.

We can expect that Iranian trout diverged significantly genetically from other trout populations of the Caspian Sea, taking into account the differences by allozyme markers, not only between the allele frequencies, but also between the allele compositions of some loci. Such investigation would be repeated, and probably the number of studied allozyme loci and the number of trout samples from Iranian rivers would be increase in order to obtain final assessment of differences and to make the decision about their taxonomic status.
